# Evaluating In-Hospital Safety and Perioperative Costs of Total Hip Arthroplasty in Super-Elderly Patients: A Nationwide Propensity Score–Matched Analysis in Japan

**DOI:** 10.3390/jcm14217803

**Published:** 2025-11-03

**Authors:** Hidetatsu Tanaka, Kunio Tarasawa, Yu Mori, Ryuichi Kanabuchi, Kazuyoshi Baba, Hiroaki Kurishima, Kiyohide Fushimi, Kenji Fujimori, Toshimi Aizawa

**Affiliations:** 1 Department of Orthopaedic Surgery, Tohoku University Graduate School of Medicine, 1-1 Seiryo-machi, Aoba-ku, Sendai 980-8574, Miyagi, Japan; hidetatsu.tanaka.c7@tohoku.ac.jp (H.T.); ryuichi.kanabuchi.b8@tohoku.ac.jp (R.K.); kazuyoshi.baba.e3@tohoku.ac.jp (K.B.); toshimi.aizawa.a5@tohoku.ac.jp (T.A.); 2 Department of Health Administration and Policy, Tohoku University Graduate School of Medicine, 2-1 Seiryo-machi, Aoba-ku, Sendai 980-8574, Miyagi, Japan; kunio.tarasawa.b6@tohoku.ac.jp (K.T.); kenji.fujimori.e5@tohoku.ac.jp (K.F.); 3 Department of Orthopaedic Surgery, Japanese Red Cross Sendai Hospital, 2-43-3, Yagiyama hon-cho, Taihaku-ku, Sendai 982-8501, Miyagi, Japan; marronile@gmail.com; 4 Department of Health Policy and Informatics, Institute of Science Tokyo, 1-5-45 Yushima, Bunkyo-ku, Tokyo 113-8519, Japan; kfushimi.hci@tmd.ac.jp

**Keywords:** super-elderly, total hip arthroplasty, postoperative complications, nationwide database

## Abstract

**Background:** This study aimed to evaluate short-term outcomes, including in-hospital complications, mortality, and medical costs, after total hip arthroplasty (THA) in super-elderly patients aged ≥85 years compared with elderly patients aged 70–84 years, using a nationwide database in Japan. **Materials and Methods:** We conducted a retrospective cohort study using the Japanese Diagnosis Procedure Combination (DPC) database from 2011 to 2023. Patients undergoing unilateral THA were divided into super-elderly and elderly groups. Propensity score matching (1:1) was performed based on demographics and comorbidities, including Charlson Comorbidity Index (CCI). Primary outcomes included in-hospital complications and mortality; secondary outcomes included hospital length of stay, Barthel Index, and medical costs calculated on a fee-for-service basis for the perioperative period (surgery day through postoperative day 7). **Results:** A total of 11,997 matched pairs were analyzed. The super-elderly group had significantly higher rates of cerebrovascular events (0.6% vs. 0.3%; OR: 2.125; 95% CI: 1.403–3.219) and in-hospital mortality (0.2% vs. 0.0%; OR: 5.565; 95% CI: 2.106–14.71), though absolute risk differences were small (0.0029 and 0.0017, respectively). Hospital stay was longer in the super-elderly group (32.6 ± 21.3 vs. 29.5 ± 19.5 days). No significant difference in perioperative medical costs was observed between groups. **Conclusions:** Although super-elderly patients demonstrated slightly higher in-hospital risks of cerebrovascular events and mortality, the absolute risk differences were minimal. These findings suggest that elective THA can be safely performed during hospitalization in this population, although further research is needed to evaluate post-discharge outcomes.

## 1. Introduction

Total hip arthroplasty (THA) is a commonly performed, well-established procedure that provides substantial pain relief and functional restoration in patients with advanced hip disease [1]. In Japan, the increasing aging population has led to a rise in degenerative hip joint diseases, which are now a prevalent cause of hip pain among the elderly [2,3]. While life expectancy continues to rise, there is a growing reluctance among individuals to tolerate physical impairments. Consequently, more elderly patients with osteoarthritis are now considered suitable candidates for elective THA [4]. Additionally, some studies have demonstrated the utility of THA for elderly patients [5].

Postoperative complications of total hip replacement surgery in elderly patients are being investigated [6,7,8,9,10,11,12,13,14]. Some studies have reported that THA is a safe procedure even for patients aged 90 years or older, showing no increased incidence of complications despite longer hospital stays and different discharge statuses [13]. In contrast, other studies have demonstrated an age-related increase in the risk of complications [8,9,11]. Previous studies have suggested that age alone may be an independent risk factor for postoperative mortality and morbidity [15]. However, many of these studies were based on relatively small cohorts, potentially lacking sufficient statistical power and yielding inconsistent findings. Moreover, the analytical methods used may not have adequately adjusted for confounding variables, thereby limiting assessment of the independent effect of age on postoperative outcomes [6,8,13,14]. Although patients aged 90 years and above exhibited higher complication and mortality rates than younger THA recipients, their postoperative mortality remained lower than that of the general population [14]. Even though several large-scale studies exist, many have not fully considered differences in comorbidity profiles between early elderly and super-elderly populations. The detailed risks of THA in very elderly patients remain insufficiently explored, even in Japan, the country with the fastest-aging population, leaving significant knowledge gaps.

While long-term functional outcomes after THA have been well documented, evidence regarding the immediate perioperative safety in the super-elderly population remains limited. Although several Western registry analyses have reported outcomes of THA in patients aged 90 years and older, few large-scale studies have focused on Asian populations. The Japanese Diagnosis Procedure Combination (DPC) database allows detailed analysis of in-hospital complications and costs during standardized hospitalization periods [16,17,18,19,20]. Therefore, this study aimed to evaluate in-hospital safety and perioperative outcomes of THA in patients aged ≥85 years compared with those aged 70–84 years.

## 2. Materials and Methods

### 2.1. Study Design

This study utilized data from the Japanese Diagnosis Procedure Combination (DPC) database and was conducted in accordance with the ethical principles outlined in the Declaration of Helsinki. Ethical approval was obtained from the Institute of Science Tokyo (Approval No. M2000-788) and the Tohoku University Graduate School of Medicine (Approval Nos. 2021-1-1082 and 2024-1-1026).

### 2.2. Date Source

Retrospective data extraction was performed using the national administrative DPC reimbursement system database [21]. In accordance with ethical guidelines for studies using the DPC database, an opt-out approach was applied rather than obtaining individual written informed consent. Patients and their families were informed of the potential academic use of anonymized clinical data and were allowed to decline participation. No personally identifiable information was included in the dataset. All variables used in the analysis were derived from this database. The anonymized dataset included hospital identifiers, patient demographics, primary diagnoses coded using the International Classification of Diseases, 10th Revision (ICD-10), dates of admission and discharge, admission diagnoses, pre-existing comorbidities at the time of hospitalization, and in-hospital complications, all of which were recorded separately. To maintain data integrity and analytical reliability, cases with missing values in any key clinical variables were excluded. No data imputation was performed, and only complete cases were used for the final analysis.

### 2.3. Data Selection

The sample size was defined by the predetermined study period, not by an a priori power analysis. Clinical data were collected from approximately 1100 hospitals participating in the DPC system across Japan, which continuously submitted data and authorized research use throughout the study period from December 2011 to March 2023. The analysis included patients who underwent unilateral total hip arthroplasty (THA) surgery at these hospitals across Japan. Focusing on patients aged 70 years or older, this clinical study assessed postoperative complications of THA, particularly contrasting outcomes between super-elderly individuals aged 85 or older (super-elderly group) and those aged 70 to 84 (elderly group). Patient’s diagnosis with most-medical resource were included based on ICD-10 codes: osteoarthritis (OA; M160–M169), osteonecrosis of the femoral head (ONFH; M8705, M8715, M8725, M8735, M8785, M8795), and rheumatoid arthritis (RA; M0695, M0690, M0685, M0605, M0595, M0585, M0586, M1315). We excluded patients who underwent revision THA, hemiarthroplasty, hip fracture surgery, bilateral procedures within the same hospitalization, or prior ipsilateral THA. Only elective primary unilateral THA cases were included.

### 2.4. Outcomes of Interest

The primary outcomes of this study were the incidence of postoperative complications and in-hospital mortality. These outcomes were chosen for their clinical significance in assessing short-term risks following THA in elderly patients. The evaluated surgical complications included postoperative dislocation (M966, M2445, T143, T814, and T840), infection (M0095, M 0099, T793 and T845), periprosthetic fracture (S730, S7200, S7210, S7220, S7230, S7240, S7290, and T1420), nerve palsy including sciatic and femoral nerve (G570, G572, G573, G839, S740 and S750), wound dehiscence (T813), and re-operation. Hospital-acquired pneumonia, DVT, PE, cardiac events, cerebrovascular events, sepsis, acute renal failure, and in-hospital mortality were assessed as medical complications. Comorbidities were defined based on ICD-10 codes (Table S1). All outcomes were identified based on diagnostic codes. As mortality was included as an outcome, the assessment period for all complications covered the entire hospitalization.

The secondary outcome was the length of hospital stay, medical costs estimated using a fee-for-service approach for the eight days from surgery to postoperative day seven, and activities of daily living, all serving as measures of healthcare resource consumption and perioperative management demands. Hospital revenue was estimated using a fee-for-service model, as actual reimbursements to medical institutions vary based on DPC-specific adjustments and surcharges. Because hospital stays in Japan tend to be long and vary by facility, perioperative medical costs were calculated for the 8 days from the day of surgery to the seventh day after surgery.

### 2.5. Covariates

Covariates comprised age, sex, BMI, comorbid conditions such as hypertension, hyperlipidemia, diabetes mellitus, cerebrovascular disease, ischemic heart disease, chronic kidney disease, chronic pulmonary disease, liver cirrhosis, and CCI. The Barthel Index was assessed preoperatively and at discharge to evaluate activities of daily living in the elderly.

### 2.6. Propensity Score Matching

To minimize confounding in the comparison of postoperative complications between super-elderly and elderly patients, 1:1 propensity score matching was performed using clinically relevant covariates. Covariates used for confounding adjustment included sex, BMI, hypertension, hyperlipidemia, diabetes mellitus, cerebrovascular disease, ischemic heart disease, chronic kidney disease, chronic pulmonary disease, liver cirrhosis, and CCI. Propensity scores were calculated via logistic regression, and nearest-neighbor matching without replacement was conducted using a caliper width set at 0.2 times the standard deviation of the estimated propensity scores. Standardized mean differences (SMDs) were used to assess covariate balance after matching; SMDs < 0.1 were considered evidence of sufficient balance. Multivariate logistic regression analyses were conducted within the matched cohort to determine independent associations with outcomes, adjusting for the covariates included in the propensity score model. The discriminative ability of the propensity score model was evaluated using C-statistics. A receiver operating characteristic (ROC) curve was also plotted to visually assess the discrimination and calibration of the propensity score model, confirming good model performance (AUC = 0.899). This process resulted in matched cohorts for comparison between super-elderly and elderly patients.

**Figure 1:**
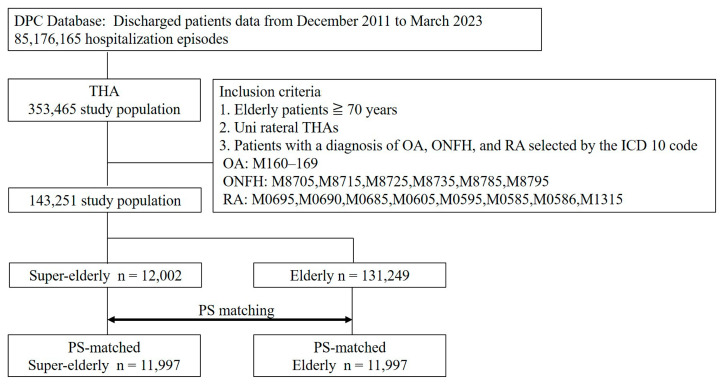
Study flow chart.

A flowchart summarizing the study design is shown in Figure 1. From the dataset spanning December 2011 to March 2023, a total of 143,251 patients met the predefined inclusion and exclusion criteria. Among them, 12,002 were categorized as super-elderly and 131,249 as elderly patients. After performing 1:1 propensity score matching based on sex, BMI, and Charlson Comorbidity Index (CCI), 11,997 matched pairs were identified from each group. Following 1:1 propensity score matching, sex, BMI, all comorbidities, and CCI achieved SMDs below 0.1, confirming adequate balance between the groups. Baseline demographic data, including age, gender, and comorbidities, for the two groups are shown in Table 1. The C-statistic for the propensity score model was 0.899, indicating good discriminatory ability. SMDs were <0.1 for all parameters. The use of cement and the administration of antiplatelet agents were more common in the super-elderly group, whereas the rate of anticoagulant agents was lower.

### 2.7. Statistical Analysis

All data are expressed as mean ± standard deviation. Significant differences between the super-elderly and elderly groups were examined using the χ^2^ test and Student’s *t*-test for each parameter. The Shapiro–Wilk test was used to evaluate normally distributed variables. Univariate logistic regression analysis was performed to evaluate severe in-hospital complications and in-hospital mortality. Multivariate logistic regression analysis was conducted to evaluate variables associated with the significant complication to identify independent risk factors. All statistical tests were two-tailed, and *p*-values < 0.001 were considered statistically significant. In addition, the standardized mean difference (SMD) between the two groups was calculated; SMDs < 0.1 were considered to indicate no statistical difference. Although SMDs indicated adequate balance between the groups after propensity score matching, a sensitivity analysis was conducted to account for the potential influence of unmeasured confounders. In this analysis, the caliper width for propensity score matching was tightened from 0.2 to 0.05, and similar risks of postoperative complications and in-hospital mortality were observed under these more stringent matching conditions, suggesting the robustness of our findings. All analyses were performed using JMP version 18.0 (SAS, Cary, North Carolina, USA).

## 3. Results

The results of the comparative analysis of complication rates between super-elderly and elderly groups are presented in Table 2. Prior to adjustment with propensity score matching, the incidence of hospital-acquired pneumonia, postoperative cerebrovascular events, acute renal failure, and in-hospital mortality was significantly higher in the super-elderly group. However, no statistically significant differences were identified between the groups for DVT, PE, cardiac events, sepsis, and surgical complications. After propensity score matching, the super-elderly group continued to show significantly higher rates of cerebrovascular events and in-hospital mortality. The corresponding risk differences (RDs) were 0.0029 (95% Confidence Interval [CI]: 0.0012 to 0.0046) for cerebrovascular events, and 0.0017 (95% CI: 0.0007 to 0.0026) for in-hospital mortality (Table 3). Although the rates of cerebrovascular events and in-hospital mortality were significantly higher in the super-elderly group (0.6% and 0.2%, respectively) compared to the elderly group (0.3% and 0.0%, respectively), the absolute risk differences were small (RD: 0.0029 and 0.0017), indicating that the clinical relevance of these findings may be limited despite statistical significance (Table 2 and Table 3).

A consistent pattern in hospital stay duration was observed before and after propensity score matching. Following matching, the super-elderly group had a longer average hospital stay (32.6 ± 21.3 days) than the elderly group (29.5 ± 19.5 days). Similarly, medical costs exhibited a comparable trend across both unmatched and matched cohorts. Although the elderly group showed slightly higher average costs, the difference was not statistically significant after matching (1,431,398 ± 412,330 vs. 1,423,705 ± 401,389, respectively). The Barthel Index also maintained a consistent trend before and after matching. Post-matching, the super-elderly group had lower mean preoperative scores (16.2 ± 5.1) than the elderly group (18.2 ± 3.8), and this difference persisted in postoperative scores as well (16.7 ± 4.4 vs. 18.4 ± 3.3, respectively) (Table 2).

The results of univariate logistic regression analyses examining the effect of being in the super-elderly group on postoperative complications and in-hospital mortality after THA are shown in Table 4. In the univariate analysis, being in the super-elderly group was significantly associated with an increased risk of cerebrovascular events and in-hospital mortality. Specifically, the super-elderly had higher odds of experiencing a cerebrovascular event (odds ratio [OR]: 2.006; 95% CI: 1.336–3.012) and in-hospital mortality (OR: 5.008; 95% CI: 1.917–13.09). For sensitivity analysis, propensity score matching with a caliper width of 0.05 yielded 11,976 matched pairs. Univariate logistic regression in this matched cohort yielded results comparable to those for postoperative complications and in-hospital mortality, reinforcing the robustness of the study findings.

The results of multivariate logistic regression analysis for cerebrovascular events and in-hospital mortality are presented in Table 5. For cerebrovascular events, the super-elderly group (OR: 2.125; 95% CI: 1.403 to 3.219) and an increase in CCI of one (OR: 2.433, 95% CI: 2.152 to 2.742) were significantly elevated risk factors. For in-hospital mortality, the super-elderly group (OR: 5.565; 95% CI: 2.106 to 14.71) and an increase in CCI of one (OR: 1.856, 95% CI: 1.398 to 2.371) were significantly elevated risk factors.

## 4. Discussion

This nationwide retrospective study using a medical claims database compared short-term postoperative complications and in-hospital mortality following THA between super-elderly patients aged ≥85 years and elderly patients aged 70–84 years. After robust adjustment through propensity score matching, the super-elderly group showed a significantly increased risk of cerebrovascular events and in-hospital mortality. Nevertheless, the absolute risk differences were minimal (0.0017–0.0029), indicating that while statistical significance was observed, the actual clinical impact of advanced age on postoperative outcomes may be modest. These results imply that super-elderly status is a detectable but relatively minor independent risk factor for adverse outcomes after THA.

These results are consistent with those reported by Vincent et al., who analyzed data from the German Total Joint Arthroplasty Registry and found that patients aged 90 years or older undergoing elective THA had significantly higher rates of in-hospital major complications and postoperative mortality than younger cohorts [14]. Notably, their study also revealed that the 1-year mortality rate in these nonagenarian THA patients was lower than that of the general population of the same age group, underscoring the importance of appropriate patient selection and perioperative optimization. The results of this study showed that although the odds ratio for mortality was high in the super-elderly, the risk difference was small and its clinical significance was considered to be limited. Although the absolute risk differences for cerebrovascular events and in-hospital mortality were small, these findings suggest that elective THA remains a feasible option for well-optimized super-elderly patients. In this fragile population, individualized perioperative risk–benefit assessment and multidisciplinary optimization are essential. Shared decision-making involving patients, families, and healthcare teams should be emphasized to ensure that surgical indications align with each patient’s overall health status, functional goals, and quality-of-life expectations.

Although the difference in Barthel Index scores between the two groups was statistically significant, it represented only a modest clinical difference. Previous rehabilitation studies in Japan have shown that a 1.7–2 point decrease in the Barthel Index corresponds to approximately a 3 to 4 day delay in achieving discharge readiness, indicating a mild delay in functional recovery among super-elderly patients. Nevertheless, this finding suggests that, when appropriately optimized and rehabilitated, elective THA remains a feasible procedure even in this fragile population.

An elevated risk of cerebrovascular complications was observed in our study population. The incidence of stroke after THA/TKA is low at 0.09%; most cases occur before discharge, with half within the second postoperative day, and elderly patients, critically ill patients, and smokers are at higher risk [22]. These findings highlight the importance of early postoperative monitoring and risk stratification to prevent cerebrovascular events in vulnerable patients.

In our study, the CCI was identified as an independent predictor for both cerebrovascular events and in-hospital mortality. This finding underscores the role of pre-existing, potentially modifiable comorbidities—particularly chronic kidney disease, congestive heart failure, and fluid and electrolyte disorders—in influencing postoperative risk [14]. Appropriate perioperative management can effectively prevent complications once they are identified, leading to improved outcomes [23]. Thus, identifying and optimizing modifiable comorbidities is key to preventing adverse postoperative outcomes [22,24]. Conversely, the super-elderly group also exhibited lower Barthel Index scores both pre- and postoperatively, reflecting greater frailty and impaired functional status, which may contribute to longer hospital stays and increased resource utilization.

The super-elderly group had a significantly longer mean length of hospital stay than the elderly group. Given the rising burden of healthcare costs and constrained funding, the cost-effectiveness of total hip arthroplasty in elderly patients has become a topic of increasing interest [22,25,26]. As hospital stays in Japan are generally longer than in many other countries due to systemic factors, we focused our cost analysis on the perioperative period (eight days). No significant elevation in costs was observed in the very elderly during this timeframe. Postoperative complications arising from pre-existing comorbidities have been linked to prolonged hospitalization, increased rates of readmission, and elevated healthcare costs, underscoring the need for preoperative patient optimization to minimize such complications [27,28]. Prehabilitation and multidisciplinary perioperative management have been reported to be key components in improving surgical outcomes among elderly patients [14]. Previous studies have demonstrated that elderly individuals benefit from prehabilitation strategies, which lead to improved postoperative outcomes not only in total joint arthroplasty but also across various surgical disciplines [29,30,31,32]. According to, preoperative optimization is expected to play a pivotal role in the future of orthopedic surgery.

The strengths of our study include its large sample size, national scope, and robust matching methodology that accounted for multiple confounders such as sex, BMI, and comorbid conditions. However, this study has several limitations. First, the analysis included only patients treated at institutions participating in the DPC database, thereby excluding those from non-DPC hospitals, which account for approximately 30% of general hospital beds in Japan. This may limit the generalizability of our findings to the entire Japanese population. Although careful propensity score matching was conducted, the influence of unmeasured confounding factors such as implant design, cementation method, blood test results, intraoperative blood loss, surgical approach, implant alignment, and rehabilitation protocol cannot be entirely ruled out. Second, because the DPC system captures only in-hospital data, long-term outcomes such as 30- or 90-day mortality, readmissions, and late complications were not available in this study. Future studies incorporating follow-up data beyond discharge are warranted to more comprehensively evaluate post-hospital outcomes. Third, potential selection bias should be acknowledged. Patients aged ≥85 years who underwent elective THA are likely to represent a relatively healthier, functionally independent subset of the general super-elderly population, potentially leading to an underestimation of true age-related risks. Nevertheless, the findings from this cohort suggest that with appropriate patient selection and perioperative preparation, elective THA can be safely performed even in the very elderly. Furthermore, because this study covered a long period from 2011 to 2023 and included data from more than 1000 hospitals, temporal and institutional variations may have influenced the outcomes. Calendar year and hospital-level effects were not modeled as fixed or random terms; therefore, standard errors may have been underestimated, and potential secular trends in practice patterns or patient selection may not have been fully captured. In addition, the cost analysis in this study was limited by the lack of adjustment for the price year and inflation rate. Medical costs were calculated based on nominal values from the DPC reimbursement database without inflation correction, and therefore do not reflect temporal changes in pricing or resource utilization during the study period. The contents of the cost calculation were confined to direct perioperative medical expenses within the first eight days after surgery. Future studies incorporating inflation-adjusted cost analyses and detailed cost component breakdowns are warranted to enhance economic comparability over time. Finally, a further limitation of this study is the lack of evaluation of long-term mortality risk after hospital discharge. Additional large-scale investigations using real-world patient data are warranted to address this issue.

## 5. Conclusions

In conclusion, elective THA in patients aged ≥85 years is associated with slightly increased risks of cerebrovascular events and in-hospital mortality, though the absolute risk remains low. These findings support the feasibility of elective THA in carefully selected super-elderly patients. Emphasis should be placed on comprehensive preoperative assessment and optimization strategies to improve outcomes in this growing patient population.

## Figures and Tables

**Table 1:** Characteristics of patients before and after propensity score matching.

	Before PS Matching			After PS Matching		
	Super-Elderly	Elderly	*p*-Value	Super-Elderly (Matched)	Elderly (Matched)	SMD
n	12,002	131,249		11,997	11,997	
Age	76.0 ± 4.1	87.0 ± 2.1	<0.001	87.0 ± 2.1	76.4 ± 4.1	
Gender						
Men	1650 (13.7%)	19,585 (14.9%)	<0.001	1649 (13.7%)	1611 (13.4%)	0.009
Women	10,352 (86.3%)	111,664 (85.1%)		10,348 (86.3%)	10,386 (86.6%)	
BMI	22.8 ± 3.5	23.8 ± 3.7	<0.001	22.8 ± 3.5	22.8 ± 3.4	0.017
Comorbidities						
Hypertension	3697 (30.8%)	31,180 (23.8%)	<0.001	3692 (30.8%)	3641 (30.4%)	0.009
Hyperlipidemia	1233 (10.3%)	12,665 (9.6%)	0.028	1232 (10.3%)	1175 (9.8%)	0.016
Diabetes	1397 (11.6%)	16,778 (12.8%)	<0.001	1397 (11.6%)	1315 (11.0%)	0.021
Cerebrovascular disease	455 (3.8%)	2964 (2.3%)	<0.001	453 (3.8%)	456 (3.8%)	0.001
Ischemic heart disease	851 (7.1%)	5281 (4.0%)	<0.001	847 (7.1%)	814 (6.8%)	0.000
Chronic renal dysfunction	336 (2.8%)	887 (0.7%)	0.861	332 (2.8%)	303 (2.5%)	0.015
Chronic lung disease	79 (0.7%)	1925 (1.5%)	<0.001	79 (0.7%)	65 (0.5%)	0.000
Liver cirrhosis	270 (2.2%)	2759 (2.1%)	0.289	270 (2.2%)	229 (1.9%)	0.101
CCI	0.6 ± 0.9	0.5 ± 0.9	<0.001	0.6 ± 0.9	0.6 ± 1.0	0.029
			*p*-value			*p*-value
Use of bone cement	2668 (22.2%)	24,608 (18.7%)	<0.001	2665 (22.2%)	2368 (19.7%)	<0.001
Medications						
Anticoagulant agent	8056 (67.1%)	97,214 (84.0%)	<0.001	8052 (67.1%)	8734 (72.8%)	<0.001
Antiplatelet agent	1981 (16.5%)	13,307 (10.1%)	<0.001	1978 (16.5%)	1447 (12.1%)	<0.001

Age, BMI, CCI, and Length of stay are shown as mean ± standard deviation. *p*-values of <0.001 are considered significant by Student’s *t*-test and χ^2^ test. PS means propensity score, SMD means standard mean difference, BMI means body mass index, and CCI means Charlson comorbidity index.

**Table 2:** Comparison of surgical and medical complications before and after propensity score matching.

	Before PS Matching			After PS Matching		
	Super-Elderly	Elderly	*p*-Value	Super-Elderly (Matched)	Elderly (Matched)	*p*-Value
Dislocation	121 (1.0%)	1025 (0.8%)	0.101	121 (1.0%)	107 (0.9%)	0.387
Infection	83 (0.7%)	1063 (0.8%)	0.185	83 (0.7%)	109 (0.9%)	0.070
Periprosthetic fracture	77 (0.6%)	625 (0.5%)	0.166	77 (0.6%)	74 (0.6%)	0.870
Nerve palsy	9 (0.1%)	70 (0.1%)	0.309	9 (0.1%)	10 (0.1%)	0.999
Wound dehiscence	14 (0.1%)	147 (0.1%)	0.886	14 (0.1%)	15 (0.1%)	0.999
Reoperation	201 (1.6%)	2026 (1.5%)	0.264	201 (1.6%)	213 (1.8%)	0.586
Hospital-acquired pneumonia	46 (0.4%)	204(0.2%)	<0.001	46 (0.4%)	27 (0.2%)	0.034
DVT	704 (5.9%)	7366 (5.6%)	0.247	704 (5.9%)	674 (5.6%)	0.421
PE	33 (0.3%)	261 (0.2%)	0.091	33 (0.3%)	34 (0.3%)	0.999
Cardiac event	6 (0.1%)	28 (0.0%)	0.061	6 (0.1%)	5 (0.0%)	0.999
Cerebrovascular event	70 (0.6%)	315 (0.2%)	<0.001	70 (0.6%)	35 (0.3%)	<0.001
Sepsis	70 (0.6%)	821 (0.6%)	0.627	70 (0.6%)	84 (0.7%)	0.293
Acute renal failure	12 (0.1%)	35 (0.0%)	<0.001	12 (0.1%)	2 (0.0%)	0.013
Mortality during hospitalization	25 (0.2%)	71 (0.1%)	<0.001	25 (0.2%)	5 (0.0%)	<0.001
Length of stay	32.6 ± 21.3	28.5 ± 17.3	<0.001	32.6 ± 21.3	29.5 ± 19.5	<0.001
Medical costs (yen)	1,423,694 ± 412,256	1,433,864 ± 400,044	0.010	1,423,705 ± 401,389	1,431,398 ± 412,330	0.072
Preoperative BI	16.2 ± 5.0	18.5 ± 3.5	<0.001	16.2 ± 5.1	18.2 ± 3.8	<0.001
Postoperative BI	16.7 ± 4.4	18.6 ± 3.1	<0.001	16.7 ± 4.4	18.4 ± 3.3	<0.001

*p*-values of <0.001 are considered significant by the χ^2^ test. PS means propensity score, DVT means Deep Vein Thrombosis, and PE means Pulmonary Embolism.

**Table 3:** Risk differences in postoperative complications associated with super-elderly before and after propensity score matching.

	Before PS Matching			After PS Matching		
	Risk Difference	95% CI	*p*-Value	Risk Difference	95% CI	*p*-Value
Dislocation	0.0023	0.0004 to 0.0041	0.101	0.0012	−0.0013 to 0.0036	0.387
Infection	−0.0012	−0.0027 to 0.0004	0.185	−0.0022	−0.0044 to 0.000	0.070
Periprosthetic fracture	0.0017	0.0002 to 0.0031	0.166	0.0003	−0.0018 to 0.0023	0.870
Nerve palsy	0.0002	−0.0003 to 0.0007	0.309	0.0000	−0.0008 to 0.0006	0.999
Wound dehiscence	0.0000	−0.0006 to 0.0007	0.886	0.0000	−0.0001 to 0.0008	0.999
Reoperation	0.0013	−0.0011 to 0.0037	0.264	−0.001	−0.0043 to 0.0023	0.586
Hospital-acquired pneumonia	0.0023	0.0012 to 0.0034	<0.001	0.0016	0.0002 to 0.0030	0.034
DVT	0.0025	−0.0019 to 0.0069	0.247	0.0025	−0.0034 to 0.0084	0.421
PE	0.0008	−0.0002 to 0.0017	0.091	0.0000	−0.0014 to 0.0012	0.999
Cardiac event	0.0003	−0.0001 to 0.0007	0.061	0.0000	−0.0005 to 0.0006	0.999
Cerebrovascular event	0.0034	0.0020 to 0.0048	<0.001	0.0029	0.0012 to 0.0046	<0.001
Sepsis	−0.0004	−0.0019 to 0.0010	0.627	−0.0012	−0.0032 to 0.0009	0.293
Acute renal failure	0.0007	0.0002 to 0.0013	<0.001	0.0008	−0.002 to 0.001	0.013
Mortality during hospitalization	0.0015	0.0007 to 0.0024	<0.001	0.0017	0.0007 to 0.0026	<0.001

*p*-values of <0.001 are considered significant by the χ^2^ test. PS means propensity score, DVT means Deep Vein Thrombosis, and PE means Pulmonary Embolism.

**Table 4:** Univariate logistic regression analysis of postoperative complications associated with super-elderly after propensity score matching using two different caliper widths for sensitivity analysis.

	Caliper Widths of 0.2			Caliper Widths of 0.05		
	OR	95% CI	*p*-Value	OR	95% CI	*p*-Value
Dislocation	1.132	0.872 to 1.470	0.387	1.461	0.989 to 2.156	0.056
Infection	0.760	0.570 to 1.012	0.070	0.720	0.433 to 1.200	0.203
Periprosthetic fracture	1.041	0.756 to 1.433	0.870	1.151	0.815 to 1.627	0.240
Nerve palsy	0.900	0.366 to 2.215	0.999	0.886	0.359 to 2.185	0.792
Wound dehiscence	0.933	0.450 to 1.934	0.999	0.992	0.470 to 2.062	0.982
Reoperation	0.943	0.776 to 1.145	0.586	0.768	0.565 to 1.043	0.09
Hospital-acquired pneumonia	1.706	1.060 to 2.746	0.034	1.538	0.945 to 2.496	0.077
DVT	1.047	0.939 to 1.168	0.421	1.054	0.945 to 1.175	0.345
PE	0.971	0.601 to 1.568	0.999	0.923	0.570 to 1.496	0.746
Cardiac event	1.200	0.366 to 3.933	0.999	1.174	0.358 to 3.854	0.791
Cerebrovascular event	2.006	1.336 to 3.012	<0.001	1.998	1.330 to 3.000	<0.001
Sepsis	0.832	0.606 to 1.144	0.293	1.112	0.633 to 1.952	0.712
Acute renal failure	6.005	1.344 to 26.84	0.013	5.654	1.257 to 25.43	0.007
Mortality during hospitalization	5.008	1.917 to 13.09	<0.001	4.611	1.750 to 12.15	<0.001

*p*-values of <0.001 are considered significant by the χ^2^ test. DVT means Deep Vein Thrombosis, and PE means Pulmonary Embolism.

**Table 5:** Multivariate logistic analysis of risk factors for Cerebrovascular event and mortality during hospitalization.

	Cerebrovascular Event				Mortality During Hospitalization			
Variable	OR	95% CI	χ^2^ Statics	*p*-Value	OR	95% CI	χ^2^ Statics	*p*-Value
Gender (Female)	1.228	0.754 to 1.999	0.682	0.409	0.777	0.304 to 1.988	0.265	0.607
BMI	1.040	0.985 to 1.097	2.046	0.153	0.828	0.735 to 0.927	11.14	<0.001
Super-elderly	2.125	1.403 to 3.219	12.67	<0.001	5.565	2.106 to 14.71	16.300	<0.001
Hypertension	1.151	0.756 to 1.734	0.43	0.512	0.769	0.333 to 1.775	0.392	0.531
Hyperlipidemia	1.520	0.866 to 2.671	2.124	0.145	0.692	0.160 to 3.000	0.267	0.606
Diabetes	0.379	0.210 to 0.682	10.44	0.001	0.428	0.122 to 1.494	2.118	0.146
Cerebrovascular disease	0.702	0.318 to 1.548	0.769	0.381	0.514	0.068 to 3.890	0.507	0.477
Ischemic heart disease	1.737	0.989 to 3.050	3.686	0.055	3.274	1.313 to 8.165	5.125	0.024
Chronic renal dysfunction	0.423	0.190 to 0.942	7.209	0.007	1.018	0.271 to 3.825	0.001	0.979
Chronic lung disease	0.000	-	0.000	0.993	4.821	1.048 to 22.17	2.861	0.091
Liver cirrhosis	0.454	0.014 to 1.493	1.692	0.193	0.000	-	3.127	0.077
CCI	2.433	2.152 to 2.742	208.3	<0.001	1.856	1.398 to 2.371	15.07	<0.001

*p*-values of <0.001 are considered significant by the χ^2^ test. OR means Odds Ratio, CI means confidence interval, and CCI means Charlson comorbidity index.

## Data Availability

The datasets generated and/or analyzed during this study are not publicly available due to their use in other projects, but are available from the corresponding author on reasonable request.

## Supplementary Materials

The following supporting information can be downloaded at: https://www.mdpi.com/article/10.3390/jcm14217803/s1, Table S1: ICD-10 code of Outcome measures.

## Abbreviations

The following abbreviations are used in this manuscript:

THA	total hip arthroplasty
DPC	Diagnosis Procedure Combination
CI	Confidence interval
PS	Propensity score
BMI	body mass index
CCI	Charlson Comorbidity Index
DVT	deep vein thrombosis
PE	pulmonary embolization
SDs	standard deviations
SMD	Standardized mean differences
OR	odds ratios

## References

[1] Learmonth I.D., Young C., Rorabeck C. (2007). The operation of the century: Total hip replacement. Lancet.

[2] Iidaka T., Horii C., Muraki S., Oka H., Kawaguchi H., Nakamura K., Akune T., Tanaka S., Yoshimura N. (2022). Trends in prevalence of hip osteoarthritis over a 10-year period in Japan: The ROAD study 2005–2015. Osteoarthr. Cartil. Open.

[3] Sato T., Yamaguchi R., Utsunomiya T., Inaba Y., Ike H., Kinoshita K., Doi K., Kawano T., Shiomoto K., Hara T. (2025). Etiology and clinical trends in hip osteoarthritis in Japan: Insights from a multicenter cross-sectional study. J. Orthop. Sci..

[4] Rupp M., Lau E.M., Kurtz S.M., Alt V. (2020). Projections of Primary TKA and THA in Germany from 2016 Through 2040. Clin. Orthop. Relat. Res..

[5] Anderson P.M., Vollmann P., Weissenberger M., Rudert M. (2022). Total hip arthroplasty in geriatric patients—A single-center expe-rience. SICOT J..

[6] Alfonso D.T., Howell R.D., Strauss E.J., Di Cesare P.E. (2007). Total hip and knee arthroplasty in nonagenarians. J Arthroplast..

[7] Dorman T., Adamczyk E., Synder M., Sibiński M. (2008). Elective total hip arthroplasty for patients 75 years of age and older. Chir. Narzadow Ruchu Ortop Pol..

[8] Murphy B.P., Dowsey M.M., Choong P.F. (2018). The Impact of Advanced Age on the Outcomes of Primary Total Hip and Knee Arthroplasty for Osteoarthritis: A Systematic Review. JBJS Rev..

[9] Smith E.L., Dugdale E.M., Tybor D., Kain M. (2018). Comparing Inpatient Complication Rates between Octogenarians and Nonagenarians following Primary and Revision Total Knee Arthroplasty in a Nationally Representative Sample, 2010–2014. Geriatrics.

[10] Sherman A.E., Plantz M.A., Hardt K.D. (2020). Outcomes of Elective Total Hip Arthroplasty in Nonagenarians and Centenarians. J. Arthroplast..

[11] Yohe N., Weisberg M.D., Ciminero M., Mannino A., Erez O., Saleh A. (2020). Complications and Readmissions After Total Hip Re-placement in Octogenarians and Nonagenarians. Geriatr. Orthop. Surg. Rehabil..

[12] Gomez Alcaraz J., Pardo Garcia J.M., Sevilla Fernandez J., Delgado Diaz E., Moreno Beamud J.A. (2021). Primary total hip arthroplasty in elderly patients over 85 years old: Risks, complications and medium-long term results. Rev. Esp. Cir. Ortop. Traumatol. (Engl. Ed.).

[13] Kurapatti M., Patel V., Arraut J., Oakley C., Rozell J.C., Schwarzkopf R. (2023). Primary total hip arthroplasty in patients older than 90 years of age: A retrospective matched cohort study. HIP Int..

[14] Leopold V.J., Krull P., Hardt S., Hipfl C., Melsheimer O., Steinbrück A., Giebel G.M. (2023). Is Elective Total Hip Arthroplasty Safe in Nona-genarians?: An Arthroplasty Registry Analysis. J. Bone Joint Surg. Am..

[15] Nanjayan S.K., Swamy G.N., Yellu S., Yallappa S., Abuzakuk T., Straw R. (2014). In-hospital complications following primary total hip and knee arthroplasty in octogenarian and nonagenarian patients. J. Orthop. Traumatol..

[16] Mori Y., Tarasawa K., Tanaka H., Kamimura M., Harada K., Mori N., Fushimi K., Aizawa T., Fujimori K. (2025). Thromboembolic and infectious complication risks in TKA and UKA: Evidence from a Japanese nationwide cohort. Knee Surg. Relat. Res..

[17] Mori Y., Tarasawa K., Tanaka H., Kanabuchi R., Kuriyama Y., Hatakeyama H., Mori N., Fushimi K., Aizawa T., Fujimori K. (2025). Increased early complication rates following total hip arthroplasty in rheumatoid arthritis patients based on a Japanese nationwide medical claims database study. Sci. Rep..

[18] Mori Y., Tarasawa K., Tanaka H., Mori N., Fushimi K., Aizawa T., Fujimori K. (2025). Nationwide database study of postoperative sequelae and in-hospital mortality in super-elderly hip fracture patients. J. Bone Miner. Metab..

[19] Tanaka H., Tarasawa K., Mori Y., Fushimi K., Fujimori K., Aizawa T. (2025). Increased complications of proximal femur fractures during the COVID-19 pandemic: A nationwide medical claims database study in Japan. J. Bone Miner. Metab..

[20] Tanaka H., Tarasawa K., Mori Y., Kuriyama Y., Kawamata H., Fushimi K., Fujimori K., Aizawa T. (2025). Does Osteonecrosis of the Femoral Head Increase Early Complication Rates After Total Hip Arthroplasty? A Japanese Nationwide Medical Claims Database Study. J. Arthroplast..

[21] Matsuda S. (2016). Development of Case Mix Based Evaluation System in Japan. Jpn. Hosp..

[22] Kleiboer B., Layer M.A., Cafuir L.A., Cuker A., Escobar M., Eyster M.E., Kraut E., Leavitt A.D., Lentz S.R., Quon D. (2022). Postoperative bleeding complications in patients with hemophilia undergoing major orthopedic surgery: A prospective multicenter observational study. J. Thromb. Haemost..

[23] Antonelli B., Chen A.F. (2019). Reducing the risk of infection after total joint arthroplasty: Preoperative optimization. Arthroplasty.

[24] Craig R.G., Hunter J.M. (2008). Recent developments in the perioperative management of adult patients with chronic kidney disease. Br. J. Anaesth..

[25] Bozic K.J., Saleh K.J., Rosenberg A.G., Rubash H.E. (2004). Economic evaluation in total hip arthroplasty: Analysis and review of the literature. J. Arthroplast..

[26] Spangehl M.J. (2017). Health care economics and total joint arthroplasty. Arthroplast. Today.

[27] Jaenisch M., Wirtz D.C. (2022). Patient optimization before hip revision arthroplasty: How to handle comorbidities. Orthopadie.

[28] Ng M.K., Lam A., Diamond K., Piuzzi N.S., Roche M., Erez O., Mont M.A. (2023). What are the Causes, Costs, and Risk-Factors for Emergency Department Visits Following Primary Total Hip Arthroplasty? An Analysis of 1,018,772 Patients. J. Arthroplast..

[29] Hoogeboom T.J., Dronkers J.J., Ende C.H.v.D., Oosting E., van Meeteren N.L. (2010). Preoperative therapeutic exercise in frail elderly scheduled for total hip replacement: A randomized pilot trial. Clin. Rehabil..

[30] Oosting E., Jans M.P., Dronkers J.J., Naber R.H., Dronkers-Landman C.M., Vries S.M.A.-D., van Meeteren N.L. (2012). Preoperative home-based physical therapy versus usual care to improve functional health of frail older adults scheduled for elective total hip arthroplasty: A pilot randomized controlled trial. Arch. Phys. Med. Rehabil..

[31] Chen H., Li S., Ruan T., Liu L., Fang L. (2018). Is it necessary to perform prehabilitation exercise for patients undergoing total knee arthroplasty: Meta-analysis of randomized controlled trials. Physician Sportsmed..

[32] Jahic D., Omerovic D., Tanovic A., Dzankovic F., Campara M. (2018). The Effect of Prehabilitation on Postoperative Outcome in Patients Following Primary Total Knee Arthroplasty. Med. Arch..

