# Supplementary material for: Evaluating In-Hospital Safety and Perioperative Costs of Total Hip Arthroplasty in Super-Elderly Patients: A Nationwide Propensity Score–Matched Analysis in Japan
Source: J Clin Med. 2025 Nov 3;14(21):7803. doi: 10.3390/jcm14217803 (PMC12609524; doi:10.3390/jcm14217803)
Supplement: Supplementary file 1 [file jcm-14-07803-s001.zip › jcm-3918931-supplementary.pdf]

Table S1. ICD-10 code of Outcome measures.

**Outcome Measures**	
pneumonia	J13, J14, J150, J151, J152, J153, J154, J155, J156, J157, J158, J159, J160, J180, J181, J182, J188, J189, J202, J690, J958
DVT	I800, I801, I802
PE	I269
Cardiac event	I210, I211, I212, I213, I214, I219, I220, I221, I228, I229
Cerebrovascular event	I64, I600, I601, I602, I603, I604, I605, I606, I607, I608, I609, I610, I611, I613, I614, I615, I616, I618, I619, I620, I621, I629, I630, I631, I632, I633, I634, I635, I636, I638, I639, I650, I651, I652, I653, I660, I661, I662, I663, I668, I669, I670, I672, I673, I674, I675, I676, I677, I678, I679, I690, I691, I693, I694
Acute renal failure	N170, N171, N172, N178, N179

ICD: International Classification of Diseases, DVT: deep vein thrombosis; PE: pulmonary embolism.
